# Correction to: Cytokine storm and histopathological findings in 60 cases of COVID-19-related death: from viral load research to immunohistochemical quantification of major players IL-1β, IL-6, IL-15 and TNF-α

**DOI:** 10.1007/s12024-021-00446-1

**Published:** 2021-12-15

**Authors:** Paolo Frisoni, Margherita Neri, Stefano D’Errico, Letizia Alfieri, Diana Bonuccelli, Mariano Cingolani, Marco Di Paolo, Rosa Maria Gaudio, Maurizio Lestani, Matteo Marti, Massimo Martelloni, Carlo Moreschi, Alessandro Santurro, Matteo Scopetti, Ombretta Turriziani, Martina Zanon, Roberto Scendoni, Paola Frati, Vittorio Fineschi

**Affiliations:** 1 Department of Medical Sciences, University of Ferrara, Ferrara, Italy; 2 Department of Surgical, Medical and Health Sciences, University of Trieste, Trieste, Italy; 3 Department of Legal Medicine, Territorial Unit USL Toscana Nordovest Lucca, Pisa, Italy; 4 Department of Law, Institute of Legal Medicine, University of Macerata, Macerata, Italy; 5 Department of Surgical Pathology, Medical, Molecular and Critical Area, Institute of Legal Medicine, University of Pisa, 56126 Pisa, PI Italy; 6 Department of Translational Medicine, University of Ferrara, Ferrara, Italy; 7 Pathology Unit, Territorial Unit ULSS 7 Pedemontana, Alto Vicentino Hospital, Thiene, Italy; 8 Department of Medical Area (DAME), University of Udine, Udine, Italy; 9 Department of Anatomical, Histological, Forensic and Orthopaedic Sciences (SAIMLAL), Sapienza University of Rome, Rome, Italy; 10 Department of Molecular Medicine, Laboratory of Virology, Sapienza University of Rome, Rome, Italy

## Correction to: Forensic Science, Medicine and Pathology https://doi.org/10.1007/s12024-021-00414-9

The original version of this article unfortunately contained mistakes. Tables 1–4 are missing in the published version.

The original article has been corrected.

